# Limits of Babinet’s principle for solid and hollow plasmonic antennas

**DOI:** 10.1038/s41598-019-40500-1

**Published:** 2019-03-08

**Authors:** M. Horák, V. Křápek, M. Hrtoň, A. Konečná, F. Ligmajer, M. Stöger-Pollach, T. Šamořil, A. Paták, Z. Édes, O. Metelka, J. Babocký, T. Šikola

**Affiliations:** 1 Central European Institute of Technology, Brno University of Technology, Purkyňova 123, 612 00 Brno, Czech Republic; 2 Institute of Physical Engineering, Brno University of Technology, Technická 2, 616 69 Brno, Czech Republic; 3 Materials Physics Center CSIC-UPV/EHU, Paseo Manuel de Lardizabal 5, 20018 San Sebastián, Spain; 4 University Service Centre for Transmission Electron Microscopy, TU Wien, Wiedner Hauptstraße 8–10, 1040 Wien, Austria; 5 Institute of Scientific Instruments, Czech Academy of Sciences, Královopolská 147, 612 00 Brno, Czech Republic

## Abstract

We present an experimental and theoretical study of Babinet’s principle of complementarity in plasmonics. We have used spatially-resolved electron energy loss spectroscopy and cathodoluminescence to investigate electromagnetic response of elementary plasmonic antenna: gold discs and complementary disc-shaped apertures in a gold layer. We have also calculated their response to the plane wave illumination. While the qualitative validity of Babinet’s principle has been confirmed, quantitative differences have been found related to the energy and quality factor of the resonances and the magnitude of related near fields. In particular, apertures were found to exhibit stronger interaction with the electron beam than solid antennas, which makes them a remarkable alternative of the usual plasmonic-antennas design. We also examine the possibility of magnetic near field imaging based on the Babinet’s principle.

## Introduction

Localized surface plasmons (LSP) are self-sustained collective oscillations of free electrons in metal nano- and microstructures coupled to the local electromagnetic field^1^. The metal structures supporting LSP are often called plasmonic antennas (PAs). A characteristic feature of PAs is a strong enhancement of electromagnetic field within the surrounding dielectric together with its confinement on the subwavelength scale, which can be utilized to control various optical processes in the visible and near infrared spectral region even below the free space diffraction limit^2,3^. Easy tunability of the optical properties of nanostructures via engineering their size, shape, or dielectric environment^4^ opens wide field of applications. They include tip enhanced Raman spectroscopy^5^, single molecule fluorescence^6^, improving the efficiency of solar cells^7^, or enhancing the photoluminescence of solid state emitters^8–10^, just to name a few. New discoveries with high application potential are often connected to implementation of new concepts into the field of plasmonics. Such example is the Babinet’s principle of complementarity, which describes the correspondence between optical response of apertures and their complementary particles^11^.

In the pioneering works on Babinet’s principle in plasmonics, the main complementary characteristics of apertures and particles were identified^12–14^, and methods of their evaluation were proposed^15^. In particular, the correspondence not only between reflectance and transmittance was verified, but also between LSP resonances (LSPR). The Babinet’s principle predicts that a LSPR shall have the same spectral position (energy) in both a particle and a corresponding aperture. However, the electric and magnetic in-plane components of the near field with mutually perpendicular polarizations shall be interchanged. For example, assuming the excitation (plane wave, electron beam, etc.) propagating along the *z* direction, the *x*-polarized electric component in a particle shall have the same spatial distribution as the *y*-polarized magnetic component in the corresponding aperture (*x* and *y* denoting the orthogonal in-plane directions). Seminal work by Hentschel *et al*.^16^ provided a comprehensive survey of the important principles involved in coupled solid and hollow plasmonic nanostructures. Plasmonic nanostructures can be used to direct radiation of nanoscale emitters, which was investigated in context of Babinet’s complementarity^17^, and the results demonstrated the immense potential of hollow plasmonic antennas for influencing magnetic resonances of both artificial and natural emitters^18^. The Babinet-complementary nanostructures were also discussed from the perspective of nonlinear optical processes^19^, their sensing capabilities were evaluated both in the visible^20^ and infrared^21^, and complementary metamaterials on their basis were demonstrated^22^.

Mapping of LSP resonances in metallic nanostructures with high spatial and energy resolution is necessary to understand their optical properties. LSPR can be investigated using many techniques. Very good spatial and reasonable energy resolution is achieved by electron beam spectroscopy^23,24^, such as electron energy loss spectroscopy (EELS) and cathodoluminescence (CL). Both techniques utilize an electron beam that interacts with the metallic nanoparticle and excites LSPR. EELS measures the energy transferred from electrons to LSP^24–26^ and CL deals with the light which LSP emit during their decay^27^. Near field of plasmon resonances can be also mapped using scanning near-field optical microscopy (SNOM), which offers a sensitivity to specific near field components (in-plane and out-of-plane)^25,28–30^. Additional spatially-resolved spectroscopic techniques are available for specific spectral regions^31^. The experimental methods for investigation of LSPs are complemented by optical spectroscopy^13,32^ which offers excellent spectral resolution but no (or at best wavelength-limited) spatial resolution.

The qualitative validity of Babinet’s principle is not in question. Numerous studies demonstrated similar energies of LSPR and complementary spatial distribution of the near fields^13,33^. In these studies, minor differences in the energies of LSPR in solid and hollow PAs (particles and apertures) are often ascribed to fabrication-related differences in PAs’ size and shape. The quantitative validity of Babinet’s principle is less clear and is discussed rather infrequently^13,31,34^. Importantly, Babinet’s principle holds for an infinitely thin and perfectly opaque (*i.e*., made of ideal metal) screen^11,12^. The first requirement is easily met for long wavelengths but becomes violated for shorter wavelengths where the lateral dimensions of the PAs are comparable to their thickness (we note that the thickness about 20 nm or more ensures that the thin film exhibits the dielectric function similar to a bulk metal). Similarly, the opacity of the screen is not perfect close to the plasma frequency of the metal. Some of the recent works also relate the violation of the Babinet’s principle to the interband absorptions in the metal and related deviation of the dielectric function from the Drude model^34^. Thus, the quantitative agreement between solid and hollow PAs shall deteriorate as the wavelength decreases from THz over infrared to the visible spectral region. This is indeed confirmed by several studies. In microwave-to-THz, copper split-ring resonators^31^ with fundamental resonance frequency of 75 GHz (corresponding to the wavelength of 4 mm) exhibited nearly perfect correspondence in the energies of LSPR and transmission spectra, and also fair correspondence between the near fields (although not quantified). Zentgraf *et al*.^13^ studied infrared split-ring resonator (fundamental resonance frequency of about 70 THz corresponding to the wavelength of 4 μm). They showed good agreement in the spatial profiles of related near fields. However, the magnitude of the field in the aperture was about twice smaller than in the particle (see Fig. 4 in ref.^13^). LSP energies exhibited some differences which were not quantified. In the visible, Mizobata *et al*. studied far-field and near-field spectroscopic properties of complementary gold nanowires and nanovoids using an aperture-type SNOM^34^. They concluded that Babinet’s principle was not valid for the far-field transmission spectra, whereas the near-field extinction spectra showed nearly complementary spectral features.

The types of PAs frequently studied from the perspective of Babinet’s principle are split-ring resonators^13,31,33^ probably inspired by their role of building blocks for metamaterials, and rod-like PAs with polarization sensitive and easy-to-interpret plasmonic spectra^34–37^. The disc shape of PAs has been studied rather rarely in the context of Babinet’s principle, yet we believe it is an ideal shape for the quantitative study. It is rather easy to fabricate with no sharp edges (which might have different fabrication quality for particles and apertures). Rotational symmetry by design allows to identify fabrication imperfections. Finally, plasmonic properties of disc-shaped solid PAs are well known^38–41^ and studies for disc-shaped apertures are also available^42–44^.

In our contribution, we focus on these elementary PAs–gold discs and disc-shaped apertures in a gold layer. Our aim is to investigate the electromagnetic properties of Babinet-complementary structures and describe similarities and differences. First, we use numerical simulations to calculate the response of PAs to an electromagnetic plane wave. Next, we use EELS and CL to determine spectral position of the plasmonic modes and the spatial distribution of their local field. The experimental results are supported and interpreted with numerical simulations. To allow comparison of PAs with not exactly identical dimensions, we study the size dependence of their response. Importantly, we also compare strength of the response of complementary PAs.

## Results and Discussion

Babinet’s principle predicts complementary properties of solid and hollow PAs (particles and apertures). We anticipate two origins of violation of the Babinet’s principle resulting into non-complementary behavior of both PA types. First, Babinet’s principle holds for an infinitesimally thin and perfectly conducting metallic layer, which makes it only approximate for a real metallic PAs with finite thickness and conductivity, supported by a substrate. Second, different fabrication and operational conditions can also contribute to the non-complementary behavior. To distinguish between both effects, we first discuss the results of numerical modeling of PAs, subject only to the former effect.

### Interaction with electromagnetic plane wave

Although we use electron microscopy as our experimental technique, it is instructive to calculate the response of PA to a plane wave, in particular its absorption and scattering. The magnitude of these effects is described by the (wavelength-dependent) scattering (*C*_scat_) and absorption cross-sections (*C*_abs_), respectively, defined as the ratio of the power scattered (*P*_scat_) or absorbed (*P*_abs_) by the PA and the intensity of the impinging wave *I*_0_: *C*_scat_ = *P*_scat_/*I*_0_, *C*_abs_ = *P*_abs_/*I*_0_. We note that peak energies in the extinction (*C*_ext_ = *C*_scat_ + *C*_abs_) and scattering cross-sections represent reasonably well the LSP spectral positions in EELS and CL spectra, respectively. Further, these calculations are less complex and easier to interpret due to smoother excitation field and much weaker response of higher plasmonic modes.

**Figure 1:**
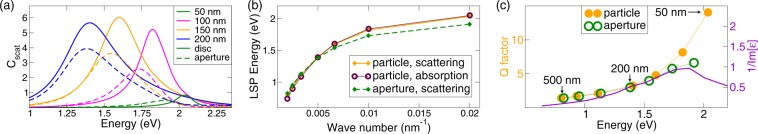
(**a**) Calculated scattering cross-section of disc-shaped plasmonic particles (solid lines) and apertures (dashed lines) for several antenna diameters. The values are normalized by the geometric cross-section (i.e., area of the disc). (**b**) Dispersion relation for LSPR: Peak energy of the calculated scattering (or absorption) cross-section as a function of a wave number represented by a reciprocal diameter. (**c**) Q factor of individual LSPR in particles (full circles) and apertures (empty circles). The lines are guides to the eye. Arrows indicate specific PA diameters, individual points from high to low energy corresponds to the diameters of 50, 100, 150, 200, 300, 400, and 500 nm. Magenta line represents the reciprocal value of the imaginary part of the dielectric function.

Scattering cross-section was calculated by FDTD (see Methods) using a plane wave for the illumination of the gold antennas with the height of 30 nm and the diameters between 50 and 500 nm. Absorption cross-section was calculated only for the particles, the calculations for the apertures did not converge. The scattering cross-sections are shown in Fig. 1(a). All scattering spectra consist of a single peak corresponding to the dipole plasmon mode. It is instructive to plot the energy of the peak as a function of inverted PA diameter, corresponding to an effective wave number [see Fig. 1(b)]^45,46^. We observe the following: (i) The energy of all peaks blue-shifts with increasing wave number (decreasing PA diameter). The blue shift saturates at the energy corresponding to the onset of interband transitions in gold. (ii) The intensity of the peaks scales with the geometrical area of the antennas, except for the smallest (deeply subwavelength) antennas, where it is considerably weaker. This is consistent with early findings of Rayleigh who showed that for small (subwavelength) scatterers the scattering cross-section goes as the sixth power of the dimension^47^. (iii) The width of the scattering peaks decreases with increasing energy (*i.e*., with decreasing diameter). The Q factor, defined as the energy of resonance divided by the full width at half maximum (FWHM) of the scattering peaks, is shown in Fig. 1(c). We note that irregular spectral profile of the peaks prevented fitting a specific function and both resonance energy and FWHM were retrieved directly from the data in Fig. 1(a). Q factor increases with increasing energy, which is attributed to a decrease in the imaginary part of the dielectric function [the reciprocal value of which is also shown in Fig. 1(c)]. For the smallest PAs there is additional increase of Q factor related to suppressed radiative losses due to reduced scattering cross section.

We will now compare the response of the particles and apertures. The energies of the peaks in scattering spectra (approximately corresponding to LSPR energies) calculated for particles and apertures [solid and dashed line in Fig. 1(b)] agree well for large PA diameters but as the diameter decreases, the scattering in the apertures becomes significantly red-shifted. Naturally, PAs with the diameter comparable to their thickness (30 nm) do not fulfill the condition of infinitesimal thickness required by the Babinet’s principle, from which they therefore deviate. For example, in PAs with the diameter of 50 nm the energy difference between LSPR in both types of PAs reaches 0.12 eV. Further, the scattering peaks of particles are more intense than those of apertures. This finding holds for the full range of PA diameters investigated here (50–500 nm). The Q factor of the particles and apertures is almost identical for PA diameters above 200 nm. Below this diameter, Q factor of the particles becomes significantly larger than that of the apertures (equal to 14 and 6.6, respectively, for the diameter of 50 nm). The increase observed for the particles can be explained by the suppressed radiative losses. While the radiative losses for the apertures are suppressed even more, the Q factor undergoes much less prominent increase, indicating a significant increase of the ohmic losses. In short, as long as the simulations are considered, apertures are worse scatterers than particles and for small PA diameters (below 200 nm) they are more lossy.

**Figure 2:**
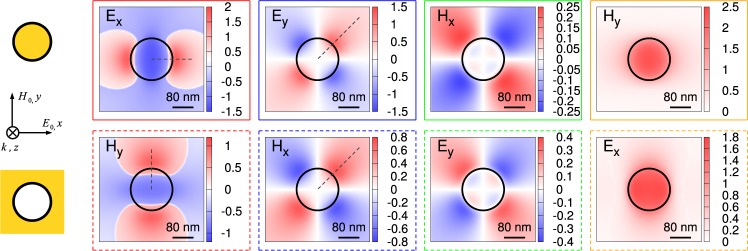
Calculated near electric and magnetic fields of disc-shaped plasmonic particles and apertures. The diameter of PAs was set to 160 nm and PAs were illuminated by *x*-polarized plane wave with the photon energy corresponding to the dipole LSPR. Field amplitudes are normalized to the incident field and multiplied by the sign of the field phase. Planar cross sections of the in-plane components of electric and magnetic scattered fields at the height of 20 nm above the PAs are shown for the particle (left column) and aperture (right column). Corresponding quantities are indicated by the frame of identical color. Black dashed lines indicate the positions of linear cross sections shown in Fig. 3.

**Figure 3:**
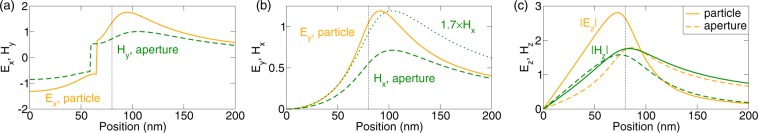
Linear cross-sections of the near field amplitudes (multiplied by the sign of phase) from Fig. 2 at the height of 20 nm above the PAs with the diameter of 160 nm. All quantities are normalized to the incident field. (**a**) In-plane components *E*_*x*_ in the particle along *x* and *H*_*y*_ in the aperture along *y*. Note that the step-like change is resulting from the step-like change of the sign of the phase, while both the amplitude and phase of the field vary smoothly. (**b**) In-plane components *E*_*y*_ in the particle and *H*_*x*_ in the aperture along *xy* diagonal. *H*_*x*_ normalized to the same amplitude as *E*_*y*_ (*i.e*., multiplied by 1.7) is shown by dotted green line. (**c**) Out-of-plane components *E*_*z*_ and *H*_*z*_. Solid (dashed) lines represent particles (apertures), orange (green) color corresponds to the electric (magnetic) field. Black dashed lines denote the PA boundary.

Figure 2 compares the calculated spatial distribution of dipolar LSP near field in a solid and a hollow PA illuminated by a plane wave polarized along the *x* direction. The diameter of the antenna is set to 160 nm. We note that the scattered fields are complex quantities determined by their magnitude and phase. In Fig. 2 we present the amplitude of the fields multiplied by the sign of the phase (+1 for the positive phase and −1 for the negative phase). Figure 2 shows a clear qualitative similarity of the spatial patterns of the field components related by the Babinet’s principle (*E*_*x*_ in the particle and *H*_*y*_ in the aperture and vice-versa, *E*_*y*_ in the particle and *H*_*x*_ in the aperture and vice-versa, related quantities are indicated by the frame of the same color). On the other hand, substantial quantitative differences in the field magnitudes are evident. Most significant differences (up to the factor of two) are found for *E*_*y*_ in the particle and *H*_*x*_ in the aperture (blue frame). Linear cross-sections shown in Fig. 3 allow for a simple comparison of the discussed near-field components. The field produced by the apertures is in general weaker than the field of the particles, in line with their weaker scattering cross sections. Similarly, the magnetic response is weaker than the electric response. This is expected, as during plasmonic oscillation the electric field energy transforms only partly into the magnetic field energy while the rest of the energy is constituted by the kinetic energy of electrons^48^. Importantly, apertures exhibit relatively stronger magnetic response than particles. Thus, one could expect that the apertures will respond more strongly to excitation with high magnetic field, such as high-energy electrons.

We also show in Fig. 3(c) the *E*_*z*_ component which is relevant for EELS and CL where it describes the interaction of a PA with the electron beam (propagating along the *z* axis^24,49^). Clearly, the maximum value of the *E*_*z*_ component in the particle is situated closer to the center of the PA than in the aperture, while the opposite is true for the *H*_*z*_ component. As both EELS and CL are sensitive to *E*_*z*_, it follows that plasmonic modes shall appear less extended in the particles than in the complementary apertures when imaged by EELS or CL.

We have also calculated EEL spectra of the complementary PAs excited by a 300-keV electron beam using finite element method software (see Methods for details). Compared to optical spectra, EELS exhibits two main differences: contribution to the loss spectra from higher order modes that are not excitable by the plane wave^46^, and, interestingly, higher loss probability in the excitation of the aperture. Although EEL spectra for the apertures were difficult to converge with respect to absolute intensity at lower energies, we obtained convergence in the peak position and also in the intensity for smaller aperture diameters.

In the following sections, we compare the theoretical results with the experimental data.

### EEL and CL spectra

**Figure 4:**
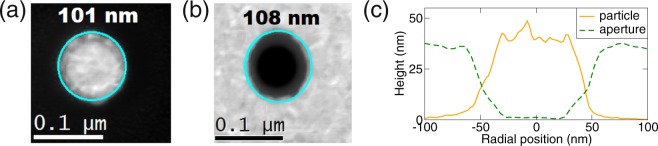
(**a**,**b**) ADF-STEM images of plasmonic antennas involved in the study: (**a**) particle with the diameter of 101 nm, (**b**) aperture with the diameter of 108 nm. White (dark) color corresponds to gold (substrate). The cyan circles show experimentally determined PA diameters, the numbers indicate the value of the diameter. (**c**) Height profiles of PAs from (**a**,**b**) obtained from EELS images.

The PAs for the EELS and CL measurement have been prepared by focused ion beam milling of 30-nm-thick gold film. Their diameter ranged from 43 nm to 164 nm. Annular dark-field (ADF) STEM images of several PAs are shown in Fig. 4(a,b). The diameter of each PA has been determined from the ADF-STEM images as the average of the diameters of the circles inscribed and circumscribed to the white/dark contrast. The error bars of the diameters in the following are obtained from the difference between the diameters of both circles. The low value of the errorbars indicates that the true PA shape can be very well approximated by a disc.

The field induced in the specimen by the probing electron acts back on the electron and decreases its energy with the probability density (so-called loss probability) reading^24,49^

1 $${{\rm{\Gamma }}}_{{\rm{EELS}}}(\omega )=\frac{e}{\pi \hslash \omega }\,\int dt{\rm{Re}}\{{e}^{-i\omega t}{\bf{v}}\cdot {{\bf{E}}}_{{\rm{ind}}}[{{\bf{r}}}_{e}(t),\omega ]\}$$

where **E**_ind_[**r**_*e*_(*t*),*ω*] is the field induced by the electron moving with the velocity **v** at the position of the electron **r**_*e*_(*t*). For the electron moving perpendicular to the sample (along the axis *z*) it is out-of-plane component of the field (*E*_*z*_) that is relevant for the interaction. Correspondence between the loss probability and the electromagnetic local density of states projected along the electron beam trajectory has been discussed^50–52^. We note that EELS is not sensitive to the in-plane field^52^. Electron losses are contributed by material-related bulk losses (proportional to the thickness of the sample) and LSP-related losses proportional to the out-of-plane field of LSPR^24,53^. To separate the contribution of LSP losses from the bulk losses, background subtraction and normalization is performed. The signal to noise ratio is better in the thinner (i.e., metal-free) areas of the specimen.

Figure 4(c) shows a cross-section of the thickness for a particle and an aperture of comparable diameters, determined by EELS in terms of inelastic mean free path (IMFP)^53^. The value of IMFP has been obtained by the mean free path estimator in software package EELSTools^54^ using the Iakoubovskii’s algorithm^55^ and for used experimental parameters it equals to 113 nm. It is evident that the edges of the PAs are sloped. This is attributed to the finite dimension of the milling beam. Further, the surface of the particle is rather humped, indicating its lower structural quality compared to the aperture. Finally, we note that the values of the thickness (ranging between 35 and 50 nm) are only approximate due to limited experimental accuracy of the EELS and uncertainty of IMFP (estimated error of 20%) and also overestimated, as the part of the apparent thickness of the PAs is related to the partly removed membrane material.

**Figure 5:**
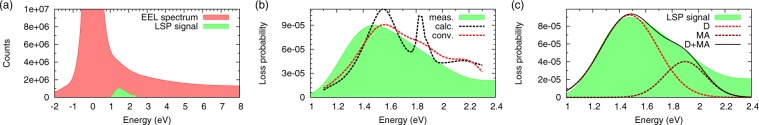
Processing of experimental EEL spectra. (**a**) Raw EEL spectrum recorded for a disc PA with a diameter of 161 nm. The spectrum is decomposed into a zero-loss peak and background (red area), and LSP-related response (green area). (**b**) Normalized LSP-related EEL spectrum (green area) compared with the calculated EEL spectrum (black dashed line) convolved with a Gaussian (full width at half maximum 0.23 eV) (red dashed line). The calculated spectra have been multiplied by a factor of 1.13 to facilitate the comparison. (**c**) Normalized LSP-related EEL spectrum (green area) decomposed into individual Gaussian peaks corresponding to the dipole mode (D, red line) and multimodal peak (MA, dark-red line). The sum of both Gaussians is shown by black solid line.

EEL and CL spectra have been measured for several disc-shaped plasmonic particles and apertures with diameters between 43 and 164 nm. Raw EEL spectra [see Fig. 5(a)] were processed by zero-loss peak and background subtraction and normalized as described in Methods. Typical resulting LSP-related loss probability spectrum (for a particle with the diameter of 161 nm) is shown in Fig. 5(b) together with a spectrum obtained from numerical simulations. While the theory predicts two distinct LSP modes (dipole and quadrupole), experimental spectrum features only single broad peak due to finite experimental resolution (mostly limited by the energy distribution of the incident electron beam). A theoretical spectrum convolved with a Gaussian of a linewidth 0.23 eV mimicking the experimental broadening [red line in Fig. 5(b)] reproduces the experimental spectrum with a reasonable accuracy. We note that the theoretical spectrum was multiplied by a factor 1.13 which equalizes its amplitude to the experimental spectrum and thus facilitates their comparison. The experimental spectrum is decomposed into individual peaks by sequential fitting of Gaussians [Fig. 5(c)]. The low-energy side of the spectrum (left side of the peak) is fitted by a single Gaussian and the residual spectrum is processed in the same way. In this way, two peaks arising from the excitation of two distinct LSP modes or their superposition can be obtained. The low-energy peak always represents the dipole LSPR. The high-energy peak, however, cannot be always related with the quadrupole LSPR as it is contributed also by the higher-order LSPRs and corresponds thus to a multimodal assembly (MA)^56^. Further, it is contributed by the bulk absorption that becomes important above 2 eV. Only for the largest PAs the high-energy peak in the experimental spectrum can be attributed to the quadrupole mode, although its linewidth is substantially exaggerated. We henceforth focus mostly on the dipole mode.

**Figure 6:**
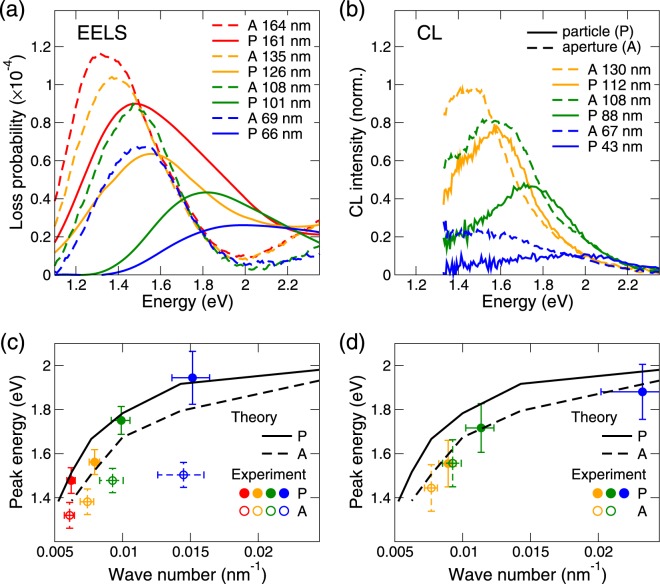
(**a**,**b**) Experimental EEL (**a**) and CL (**b**) spectra excited near the edge of the PA: particles (solid lines) and apertures (dashed lines). Specific colors correspond to similar diameters. (**c**,**d**) Dispersion relation for the dipole LSPR: calculated EEL peak energies for discs (solid line) and apertures (dashed line), data from EELS (**c**) and CL (**d**) from the top panels for the dipole LSPR in discs (filled circles) and apertures (empty circles), specific colors indicating particular spectra. We note that we use calculated EEL peak energies to represent LSPR energies in both panels. The difference between calculated CL and EEL peak energies is below 0.02 eV (1%) for discs and we anticipate similar error for apertures, where we did not perform CL calculations as they would be numerically too demanding.

Experimental EEL and CL (background-subtracted) spectra of PAs of different diameters excited near their edge are shown in Fig. 6(a,b), respectively. Within EEL spectra it is possible to identify two peaks corresponding to a dipole LSP mode and a multimodal assembly^56^. Only the dipole LSP mode is optically active and thus present in the CL spectra [Fig. 6(b)], the higher modes have no net dipole moment and are thus optically dark. The peak energies of the dipole modes are displayed in Fig. 6(c,d) in a form of dispersion relations, *i.e*., as functions of effective wave number. This wave number is equal to the reciprocal antenna diameter, determined from ADF-STEM images (the error bars correspond to circles inscribed and circumscribed to the STEM micrographs). The error bars of the energies include a standard error of the fit and a systematic error. The latter, primarily related to the background subtraction, is estimated to be 0.05 eV for EELS (see below) and 0.1 eV for CL, except for the smallest particle in EELS, where the value of 0.1 eV is taken due to flat spectrum. The dipolar peak energies determined by EELS and CL correspond to each other within the error bars. They also correspond rather well to calculations, with a slight systematic blue shift of the calculated energies that can be attributed *e.g*. to differences between real and model dielectric function of gold. The only exception is the smallest aperture (blue dashed spectra and blue empty symbols in the dispersions) for which CL features no clear peak and the peak energy determined by EELS is strongly redshifted (by 0.3 eV) from the calculated value. The origin of this difference is at present not fully clear. To rule out its relation to the incomplete zero-loss peak or background subtraction in the EEL spectra we have tested several different procedures (see Methods for details), all yielding similar LSP peak positions (within the range of about 0.05 eV). The difference might be related to the contamination of mostly organic origin evolving on the sample during its characterization in the electron microscope^57^.

Interestingly, both CL and EEL spectra are more intense for apertures than for particles. This is in agreement with numerical EEL simulations, but in contradiction with calculated optical scattering spectra [see Fig. 1(a)]. We attribute this difference to more efficient coupling of the apertures to the electromagnetic field of the relativistic electron. The ratio of the electric (*E*) and magnetic (*H*) fields for the plane wave reads ${(E/H)}_{PW}=({\mu }_{0}c)/\sqrt{{\varepsilon }_{r}}$ where *c* is the speed of light in vacuum, *μ*_0_ permeability of vacuum, and $\sqrt{{\varepsilon }_{r}}$ relative permittivity of the medium. The ratio of the radial electric field and polar magnetic field of the electron with the velocity of *v*_*E*_ reads (*E*/*H*)_*E*_ = (*μ*_0_*c*)⋅(*c*/*v*_*E*_)/*ε*_*r*_^24^. Electrons that are faster than the speed of light in the specific medium (${v}_{E} > c/\sqrt{{\varepsilon }_{r}}$) exhibit stronger magnetic field (with respect to the electric field) than the plane wave. Considering electrons with the energy of 200 keV (*v*_*E*_ ≈ 0.7*c*) and silicon nitride (*ε*_*r*_ ≈ 4) the magnetic field of electrons in our experiment is about 1.4× stronger than for the plane wave. At the same time, LSP modes in the aperture have larger magnetic-to-electric component ratio than the modes in the complementary particle (see Fig. 2). The excitation field with a larger magnetic field (relativistic electrons) thus induces more pronounced response in the apertures.

### Spatial maps of localized plasmon resonances

**Figure 7:**
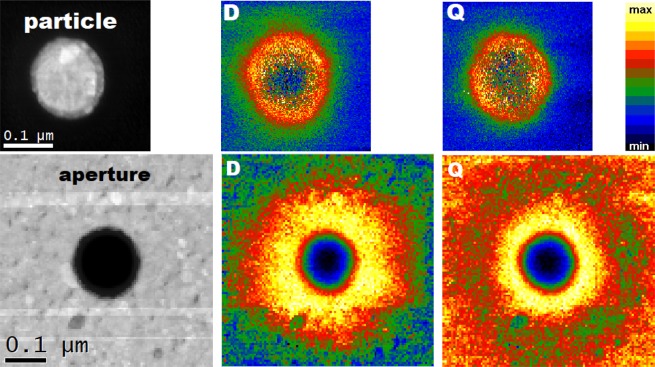
ADF-STEM images of a particle (diameter 161 nm, top) and an aperture (diameter 164 nm, bottom). Spatial maps of the loss probability for a dipole (D) and quadrupole (Q) mode.

Having identified individual plasmonic features in the EEL and CL spectra, we now discuss their spatial distribution. To this end we employed only EELS as CL suffered from the sample contamination during lengthy space-resolved measurements. The electron beam has been scanned over the sample and at each point, full spectrum was acquired. The zero-loss peak and background were not subtracted in these measurements. After the normalization, the total loss probability (including the zero-loss peak and background) integrated for the 0.1-eV-wide energy window centered around particular EEL peak has been used to represent the strength of the corresponding plasmonic mode. We stress the importance of the normalization which compensates different thickness of the parts of the sample covered and not-covered by gold. The spatial maps of the loss probability for the dipole and quadrupole mode are shown in Fig. 7 for the particle (aperture) with the diameter of 161 nm (164 nm). We note that in these large PAs the quadrupole mode is resolvable. Figure 7 clearly shows that the higher modes are more confined (they decay more rapidly from the boundary of the PA).

**Figure 8:**
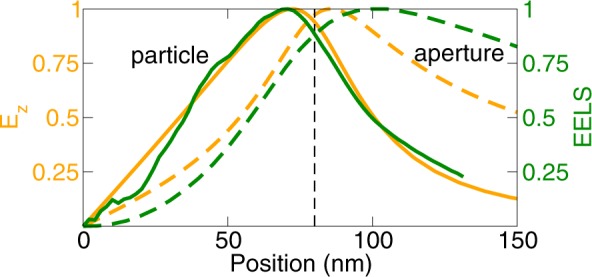
Radial distribution of the loss probability at the energy of the dipole LSP peak for a particle with a diameter of 161 nm (green solid line) and an aperture with a diameter of 164 nm (green dashed line). Experimental values are compared with the calculated results of near electric field *E*_*z*_ taken from Fig. 3(c) (orange lines). All quantities are normalized to unity and the diameters are scaled to 160 nm to allow a direct comparison of spatial profiles. Dashed line denotes the radius of the PA.

To allow for a quantitative comparison of rather noisy data, we exploited the radial symmetry and averaged the data over the angular coordinate. The radial dependences are displayed in Fig. 8 for a particle (aperture) with the diameter of 161 nm (164 nm). Clearly, the loss probability is the strongest nearby the boundary of PAs, peaking at the inner side of the boundary in the particle and at the outer side of the boundary in the aperture (*i.e*., inside metal near the boundary). This is consistent with the calculated spatial distribution of *E*_*z*_ component of the near field in the case of the plane wave illumination [*cf*. Figure 3(c)]. We note that the measured loss probability has the maximum at the distance of 71 nm (100 nm) from the center for a particle (aperture) (rescaled to the same diameter of 160 nm). Thus, the LSP resonances in the apertures are apparently more extended (*i.e*., with a maximum loss probability farther from the center of a PA) than in the particles. Qualitatively similar results were observed for all eight PAs included in the study.

Apparent larger extension of the modes in the apertures is contributed by two effects. First, electric near field of the modes is less extended (exhibits faster asymptotic decay) than the magnetic near field in the particles, while the opposite holds (in line with Babinet’s principle) in the apertures. For example, calculated near fields obtained for the plane wave illumination of a PA with the diameter of 160 nm (Fig. 3) have the following radial position (distance from the center of a PA) of the maximum values: *E*_*z*_ at 72 nm (84 nm), *H*_*z*_ at 83 nm (73 nm) for the particle (aperture). Thus, the near field components related by Babinet’s principle are equally extended. The sensitivity of EELS towards electric field component *E*_*z*_ contributes to more extended loss probability in the apertures. Further, the loss probability detected by EELS exhibits slower decay from its maximum into gold than the electric near field *E*_*z*_, which also contributes to the more extended loss probability in apertures.

In line with this finding we revisit the possibility to employ Babinet’s principle for the imaging of the near magnetic field. It has been proposed that difficult-to-access magnetic near field can be represented by the electric near field in the complementary structures^31,36^. While this approach was proven successful in THz^31^, our results indicate its rather limited applicability for the near infrared and visible spectral range and using EELS as the magnetic imaging technique. Not only the correspondence between the electric and magnetic near fields in particles and apertures is only qualitative (*cf*. Figure 3) but also experimental EELS signal does not reproduce calculated optical near field *E*_*z*_ very well.

## Conclusion

We have studied the Babinet’s complementarity for elementary disc-shaped plasmonic antennas–particles and apertures. We confirmed the qualitative validity of the Babinet’s principle–both types of plasmonic antennas exhibit LSP resonances of the comparable energy and quality factor, and the related near fields have complementary spatial distribution. On the other hand, we have found quantitative differences. Most prominent differences have been found for the near fields. In particular, the magnetic field is always weaker than its Babinet-related electric counterpart.

In general, the differences are more pronounced for the antennas with smaller diameter. This is in line with the conditions of validity of Babinet’s principle–perfectly thin and fully absorbing screen. As for the antennas with the smaller diameter, their finite thickness is more pronounced and the energy of LSPR they support is closer to the plasma frequency of the constituting metal.

Interestingly, apertures feature higher magnetic-to-electric near field ratio than particles. Consequently, apertures exhibit stronger experimental response than particles in both CL and EELS while the simulations predict weaker response to a plane electromagnetic wave (which has lower magnetic-to-electric near field ratio than relativistic electrons involved in CL and EELS).

The validity of the Babinet’s principle is relevant also for numerous applications. Babinet’s complementarity allows to toggle between the magnetic and electric response, or between the reflection and transmission mode. While particles have to be supported by the substrate, substrate-less apertures self-supported by their frame can be fabricated. Apertures also offer better heat and charge management as the thin metallic film surrounding the apertures is usually better conductor than the substrate supporting the particles.

In conclusion, solid and hollow plasmonic antennas exhibit Babinet’s complementarity. Observed differences are from the practical points of view rather minor. This opens the possibility of choice between both types of plasmonic antennas depending on desired fabrication limitations and operational conditions in a wide field of applications.

## Methods

### Fabrication of antennas

Gold disc-shaped antennas of the height of 30 nm and various diameters were prepared using focused ion beam (FIB) lithography. The silicon nitride membranes with the dimensions of 250 × 250 μm^2^ and the thickness of 30 nm (50 nm for CL studies) were used as the substrate. First, a 3-nm-thick Ti adhesion layer was deposited on the membranes, followed by the deposition of 30-nm-thick Au layer by magnetron sputtering. After that, the antennas were fabricated by focused ion beam milling (using Ga^+^ ions at 30 keV) of the gold film in a dual beam system FEI Helios. The distance between individual antennas as well as the distance of the antennas from the boundary of the metal-free square has been at least 500 nm, which is a sufficient separation to prevent the interaction between the antennas or between the antenna and the surrounding metallic frame^56^. Annular dark-field STEM images of the samples are shown in Fig. 4.

We have used thinner silicon nitride membranes for EELS than for CL. We have verified that the EEL spectra obtained for PAs on 30-nm and 50-nm-thick membranes are identical except for the lower signal-to-noise ratio of the latter ones.

Prior to the EELS and CL measurements, samples were cleaned in argon-oxygen plasma cleaner for 20 seconds and in helium-oxygen plasma cleaner for 1 minute, respectively.

### Electron energy loss spectroscopy

EELS measurements were carried out using a transmission electron microscope (TEM) FEI Titan operated at 300 kV in scanning monochromated mode. The instrument was equipped with a GIF Quantum spectrometer. The convergence semi-angle was set to 10 mrad, and the collection semi-angle to 20.5 mrad. The dispersion of the GIF was 0.01 eV per channel and the full-width-half-maximum of the zero-loss peak (ZLP) was in range from 0.13 to 0.18 eV. The probe current was adjusted around 200 pA, the acquisition time of one spectrum was in units of milliseconds to use the maximal intensity range of CCD camera in the spectrometer and avoid its overexposure. In every pixel of the spectrum image, we recorded approximately 100 spectra, which were cross-correlated and summed to reduce the noise. The spatial resolution of the EELS maps is determined by the pixel size, which was set to 2 nm in the case of disc antennas and to 5 nm in the case of apertures. Such settings led to acquiring one spectrum image with a stable electron beam in a reasonable time. To reduce noise, the spectra were integrated over pixels of spectrum images in the area where the highest excitation efficiency of the LSP is expected, *i.e*., a circular ring around the edge of the antenna. This leads to summing a large number of recorded spectra (500–5000). The spectrum was further processed by a ZLP subtraction using reflected-tail method^53^ and a background subtraction to obtain a pure LSP signal. Finally, the spectrum was divided by the integral intensity of the ZLP (the energy window for integration was from −1 eV to +1 eV) so the counts were transformed to a quantity proportional to the loss probability (referred as the loss probability in the following for simplicity). We note that the loss probability is related to the energy interval of Δ*E* = 0.01 eV, the loss probability density can be obtained by dividing the loss probability by Δ*E*.

To verify the spectra of the hollow PAs (apertures) we tested several methods for the background subtraction. The background spectrum was either modelled as exponential tail of the zero-loss peak, or determined experimentally at the membrane (pure or with the gold layer) far from the antenna. All the methods yielded comparable results with minor differences for the LSP energies below 0.05 eV.

### Cathodoluminescence spectroscopy

CL measurements were carried out using a TEM FEI Tecnai F20 operated at 200 kV in scanning mode. The instrument was equipped with a Gatan VULCAN spectrometer. The beam current was adjusted to 30 nA to get a sufficiently strong response of the antennas. Spectra were recorded in the parallel mode of the spectrometer with the spectral resolution of 3.4 nm (in wavelength, using binning 4 to reduce the noise). The acquisition time was 10 seconds. The electron beam was situated close to the edge of the antenna; the position with the highest excitation efficiency of LSP. The spectrum was background subtracted to obtain a pure LSP signal. Due to a large electron beam current, contamination was developing in course of the measurements, which prevented spatial mapping of the LSP.

### Simulations

In all simulations, the antenna has been represented by a gold disc or disc-shaped aperture (with sharp edges) of the height of 30 nm on top of 30-nm-thick silicon nitride membrane. The adhesion layer has been neglected. The dielectric function of gold was taken from ref.^58^ and the dielectric constant of the silicon nitride membrane was set equal to 4, which is a standard approximation in the considered spectral region^39^.

Scattering and absorption cross-sections and the near-field distribution has been calculated with finite-difference in time-domain (FDTD) method using a commercial software Lumerical.

Electron energy loss spectra have been calculated with finite element method (FEM) using a commercial software COMSOL Multiphysics.

## Data Availability

The datasets analysed during the current study are available from the corresponding author on reasonable request.
